# Solid Cusps

**Published:** 1896-08

**Authors:** Cephas Whitney


ARTICLE IV.

### SOLID CUSPS.

DR. CEPHAS WHITNEY.

Proceed by removing sufficient material from occluding surface of tooth, providing there is any remaining, till a piece of metal, plate No. 27, placed on cut surface, will not interfere with occlusion. This refers to the minimum cut on grinding surface only, the buccal, lingual and proximal faces being prepared as usual. Take ordinary double-pointed drawing compass (small) to measure width of band, by placing one point a little below gingival border, and the other point at buccal occlusal edge. Use this on gold plate as a gage to scribe band, much like a carpenter uses his gage.

Get your circumference, and solder in usual manner.

After fitting band to tooth, level occluding edge with fine, broad, flat file, first getting your depth with excavator while on tooth.

Seal band, and with softened modelling composition placed in same, procure bite, being careful to have patient give you all the movements of the lower jaw as in grinding. A little experience is now necessary to trim this model neatly, from an esthetic, as well as occluding standpoint; it often being advisable to change its form in small details. It may be trimmed flush with the band on its sides. With wide No. 36 or finer saw, remove model by passing saw close to leveled edge of gold.

Place pattern, cusp side up, on glass plate, oil well, surround with paper or rubber ring and run the following composition over it to a depth of about three-fourths of an inch:

| | |
|---|---|
| Plaster of Paris . . | 6 parts. |
| Plumbago . . . | 3 " |
| Asbestos (grade 3) . . | 6 " |
| Soapstone (pulv.) . | 1 " |

On removing from the ring, the composition pattern may be lifted from investment by using a pin, having previously marked the thickest place. Dry out in Lewis case heater, or, if time is limited, carry to level, soldering pad, ball up sufficient gold, same karat as band, with blow-pipe, to fill impression made by pattern, and press quickly but evenly down with large-faced hammer. You now have a *fac simile* in gold of your composition model. This solid cusp piece must be leveled on its flat surface till it is same thickness of pattern. This can be done by driving cusp side down into end wood, leaving out a little more than is sufficient to remove. The wood forms a grip, and you may now file, using an appropriate one with confidence.

The final stage is done with great ease and despatch, for, providing that you have followed instructions closely, you will find that solid cusp piece will fit leveled edge of band, coming out flush and neat at the margins. You have only to paint your borax on edge of band and flat side of cusp piece, adjust with wire or place in soldering tweezers, and solder with very high grade stuff, say two karats down, using only a small quantity.

Fine file, finishing bur, cuttle-fish, tripoli, rouge, "and there you are."

The apparent advantages of this method are these:

1st. Excellent adaptation for grinding food.

2d. Absence of any mass of inferior gold, only five or six pieces of high grade solder being used—a grain and a half for the largest crown.

3d. Freedom of solder running on to band, thus causing same to impinge on tooth.

4th. Generosity of grinding piece, thereby insuring a lasting surface, no holes appearing in a year or so from triturating, as in the ordinary crowns. A large molar crown made by this process weighing quite two pennyweights.

5th. Absolute reproduction of model, with all the fine lines. Superiority to method of driving a model into dampened asbestos, which is not only indistinct in detail, but the eminences, which are counter to fissures and depressions in model, never rise to fill same.—*Items of Interest.*

